# Cellular and molecular outcomes of glutamine supplementation in the brain of succinic semialdehyde dehydrogenase‐deficient mice

**DOI:** 10.1002/jmd2.12151

**Published:** 2020-08-15

**Authors:** Madalyn N. Brown, K. Michael Gibson, Michelle A. Schmidt, Dana C. Walters, Erland Arning, Teodoro Bottiglieri, Jean‐Baptiste Roullet

**Affiliations:** ^1^ Department of Pharmacotherapy College of Pharmacy and Pharmaceutical Sciences, Washington State University Spokane Washington USA; ^2^ Baylor Scott and White Research Institute Institute of Metabolic Disease Dallas Texas USA

**Keywords:** astrocyte, dietary supplementation, GABA, GHB, glutamine, knockout mice

## Abstract

Succinic semialdehyde dehydrogenase deficiency (SSADHD) manifests with low levels of glutamine in the brain, suggesting that central glutamine deficiency contributes to pathogenesis. Recently, we attempted to rescue the disease phenotype of *aldh5a1* ^*−/−*^ mice, a murine model of SSADHD with dietary glutamine supplementation. No clinical rescue and no central glutamine improvement were observed. Here, we report the results of follow‐up studies of the cellular and molecular basis of the resistance of the brain to glutamine supplementation. We first determined if the expression of genes involved in glutamine metabolism was impacted by glutamine feeding. We then searched for changes of brain histology in response to glutamine supplementation, with a focus on astrocytes, known regulators of glutamine synthesis in the brain. Glutamine supplementation significantly modified the expression of glutaminase (*gls*) (0.6‐fold down), glutamine synthetase (*glul*) (1.5‐fold up), and glutamine transporters (solute carrier family 7, member 5 [*slc7a5*], 2.5‐fold up; *slc38a2*, 0.6‐fold down). The number of GLUL‐labeled cells was greater in the glutamine‐supplemented group than in controls (*P* < .05). Reactive astrogliosis, a hallmark of brain inflammation in SSADHD, was confirmed. We observed a 2‐fold stronger astrocyte staining in mutants than in wild‐type controls (optical density/cell were 1.8 ± 0.08 in *aldh5a1* ^*−/−*^ and 0.99 ± 0.06 in *aldh5a1* ^*+/+*^; *P* < .0001), and a 3‐fold higher expression of *gfap* and *vimentin*. However, glutamine supplementation did not improve the histological and molecular signature of astrogliosis. Thus, glutamine supplementation impacts genes implicated in central glutamine homeostasis without improving reactive astrogliosis. The mechanisms underlying glutamine deficiency and its contribution to SSADHD pathogenesis remain unknown and should be the focus of future investigations.

**Synopsis**

Glutamine supplementation does not rescue central glutamine deficiency and astrogliosis in experimental succinic semialdehyde dehydrogenase deficiency despite significant changes in glutamine transporter gene expression.

## 1 INTRODUCTION

Succinic semialdehyde dehydrogenase deficiency (SSADHD) is a rare, genetic disease affecting γ‐aminobutyric acid (GABA) metabolism. Biochemical hallmarks include GABA and its metabolite γ‐hydroxybutyrate (GHB), which can be detected in patient blood and urine. SSADHD patients exhibit a broad range of symptoms, including developmental delay, intellectual disability, motor function disorder, and seizures, resulting in a nonspecific clinical presentation and precluding early clinical diagnosis. Current research of SSADHD has focused on identifying additional biomarkers, including decreased glutamine in humans and the murine model, aldehyde dehydrogenase 5 family member a1 knockout mice (*aldh5a1* ^−/−^).

**FIGURE 1:**
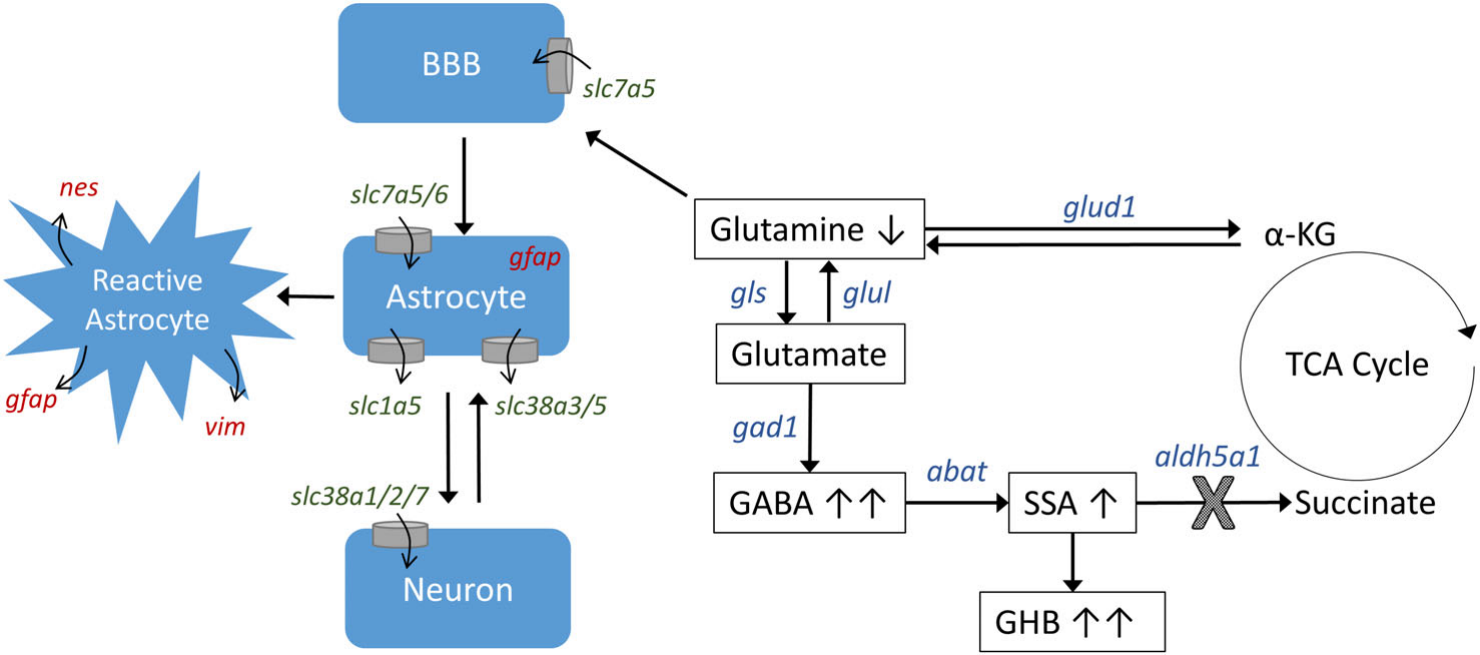
Glutamine metabolism and transport in brain. Arrows adjacent to metabolites indicate expression in SSADHD. Glutamine and GABA‐metabolites: GABA, γ‐aminobutyric acid; SSA, succinic semialdehyde; GHB, γ‐hydroxybutyrate; α‐KG, α‐ketoglutarate. Genes involved in GABA metabolism (shown in blue): *glud1*, glutamate dehydrogenase; *gls*, glutaminase; *glul*, glutamate‐ammonia ligase (glutamine synthetase); *gad1*, glutamate decarboxylase; *abat*, 4‐aminobutyrate aminotransferase (GABA transaminase); *aldh5a1*, aldehyde dehydrogenase family 5, subfamily A1 = SSADH (“X” indicates site of defect in SSADHD). Genes involved in glutamine transport (and respective protein transporter; shown in green): solute carrier family: *slc1a5* = ASCT2; *slc7a5* = LAT1; *slc7a6* = γ + LAT2; *slc38a1/2/3/5/7* = SNAT1/2/3/5/7. Reactive astrocyte/astrogliosis markers (red): *gfap* = glial fibrillary acidic protein; *nes* = nestin; *vim* = vimentin. BBB, blood‐brain barrier; SSADH, succinic semialdehyde dehydrogenase; SSADHD, succinic semialdehyde dehydrogenase deficiency; TCA cycle, tricarboxylic acid cycle = Krebs cycle

Glutamine, a nonessential amino acid, is the biological precursor of glutamate and GABA, two major neurotransmitters in the mammalian central nervous system.^1^, ^2^, ^3^ Several studies have confirmed the presence of low concentrations of glutamine in the SSADHD brain^4^, ^5^ raising the possibility that central glutamine deficiency plays a role in the pathogenesis of the disease. We attempted to address this question in experimental SSADHD mice with a study in which glutamine was provided orally via a 4% glutamine‐rich diet from conception (maternal exposure) to postweaning (30 days of life) stage.^6^ We found and reported that glutamine supplementation did not improve brain glutamine deficiency despite evidence of systemic improvement of glutamine metabolism. Glutamine supplementation did not improve behavioral deficits, nor did it rescue the short lifespan and runted bodyweight of the *aldh5a1* ^*−/−*^ mice. These findings combined raised the possibility that molecular responses to glutamine supplementation, especially those involved in glutamine metabolism and transport in the brain, might have prevented brain glutamine levels from increasing toward control values. Furthermore, since brain astrocytes play a pivotal role in maintaining glutamine and GABA metabolic homeostasis, we wondered if glutamine supplementation might have impacted astrocyte numbers or morphology to potentially offset the dietary glutamine challenge. To test these hypotheses, we used brain tissues collected as part of the study reported in Reference 6. In one set of experiments, we measured the expression of genes known to control central glutamine and GABA homeostasis (Figure 1). In another set of experiments, we used immunohistochemistry and specific astrocyte molecular markers, to assess potential changes in the cellular composition of the *aldh5a1* ^*−/−*^ mouse brain in response to glutamine supplementation. The results of these investigations show that the lack of response of central glutamine deficiency in SSADHD to dietary glutamine supplementation cannot be conclusively explained by diet‐induced changes in gene expression in the mutant brain. Our findings further suggest that reactive astrogliosis and overall brain inflammation may be potential mechanisms underlying resistance to treatment.

## 2 METHODS

### 2.1 Animals and experimental design

The experimental design of the study has been reported previously^6^ and was approved by the institutional animal care and use committee of Washington State University (ASAF 4232; 6134). Briefly, heterozygote (*aldh5a1* ^*+/−*^) breeder pairs were set up and fed either a control (CD) or glutamine‐supplemented diet (GD; 4% glutamine) for 10 days prior to conception, and the entire length of the pre‐, peri‐, and postnatal periods. *Aldh5a1* ^*−/−*^ and *aldh5a1* ^*+/+*^ offspring were assessed three times per week for body weight and food consumption. Cages were monitored daily for deceased animals. Weaned offspring were sacrificed at postnatal day (P) 30. The brain was quickly excised and cut in two halves along the midsagittal plane. Half of the brain was flash frozen in liquid nitrogen and used for gene expression analyses. The other half was processed as described in Section 3.

### 2.2 Gene expression

Frozen brain tissue was ground via mortar and pestle submersed in liquid nitrogen. The RNeasy Plus Mini Kit (Qiagen) was used for preparation and purification of RNA. RNA concentrations were quantified using Nanodrop, and cDNA was prepared using an RT kit (Qiagen). Ten nanograms of subsequent cDNA was used for each quantitative reverse transcription polymerase chain reaction. We examined gene expression using prevalidated primer sets (QuantiTech, Inc., Huntsville, Alabama; see catalog number listed below). For relative mRNA expression, genes of interest were normalized to the housekeeping gene phosphoglycerate kinase 1 (*pgk1*; cat. # QT01780331). Data points that fell outside 1.5 × the interquartile range were considered outliers and excluded prior to statistical analysis.^7^, ^8^ Three groups of genes were selected for analyses: (a) genes involved in the regulation of GABA and glutamine metabolism—*abat* (cat. # QT00152768), *aldh5a1* (cat. # QT00116333), *gad1* (cat. # QT00163527), *glud1* (cat. # QT00103411), *gls* (cat. # QT01060234), and *glul* (cat. # QT01062306); (b) glutamine transporter genes—*slc1a5* (cat. # QT00095277), *slc7a5* (cat. # QT01044932), *slc7a6* (cat. # QT00126462), *slc38a1* (cat. # QT00129878), *slc38a2* (cat. # QT00129542), *slc38a3* (cat. # QT01041544), *slc38a5* (cat. # QT00108962), and *slc38a7* (cat. # QT01073625); and (c) marker genes for astrogliosis—*gfap* (cat. # QT00101143), *nes* (cat. # QT00316799), and *vim* (cat. # QT00159670) (Figure 1).

### 2.3 Brain histology

Brain samples were fixed in 4% paraformaldehyde in phosphate‐buffered saline (PBS) for 2 days and immersed in 30% sucrose in PBS until sectioning.^9^, ^10^ Fixed samples were embedded in Tissue‐Tek optimal cutting temperature compound (Sakura Finetek USA, Inc., Torrance, California), then sliced into 20‐μm sections using a cryostat (Leica CM1950, Leica Biosystems, Buffalo Grove, Illinois), and stained for glial fibrillary acidic protein (GFAP, 1:3000 dilution, rabbit polyclonal antibody, Thermo Fisher Scientific, Waltham, Massachusetts, cat. # PA1‐10019) or glutamine synthetase (GLUL, 2 μg/mL dilution, ABfinity rabbit monoclonal antibody, Thermo Fisher Scientific, cat. # 701989). Sections were pretreated with 3% H_2_O_2_ and 0.5% Triton X in PBS, rinsed 3× with PBS, then treated with Avidin and d‐Biotin blocking solutions (AVIDIN/BIOTIN Blocking Kit, Life Technologies Corp., Frederick, Maryland, Cat #004303). Further blocking was performed with 10% goat serum in PBS + 0.3% Triton X‐100 for 1 hour at 22°C. Sections were incubated with the primary antibody (GFAP or GLUL) overnight at 22°C. After a brief rinse in PBS, sections were incubated with the secondary antibody (1:1000, biotinylated goat anti‐rabbit IgG, Vector Laboratories, Inc., Burlingame, California, cat. # BA‐1000) for 90 minutes. After three rinses with PBS, antigen‐antibody complexes were detected using VECCTASTAIN Elite ABC‐HRP kit (Vector Laboratories, Inc., cat. # PK‐6100) and DAB peroxidase substrate (1% 3,3′‐diaminobenzidine, Sigma, cat. # D8001, followed by 0.3% H_2_O_2_ in water). Sections were then mounted on glass slides, costained with 0.25% Eosin Y and dehydrated before imaging. GLUL‐labeled cells were manually counted in layer 1 of the anterior cingulate and the primary motor and somatosensory areas of the cerebral cortex, where glutamine synthetase is widely expressed. Cells were randomly counted in three separate areas of the cortex at ×10 magnification and the sum of these three cell counts was calculated.

### 2.4 Quantification of gliosis

Brain hippocampal regions (primarily CA1, CA3, and dentate gyrus) were imaged at ×40 magnification using a Zeiss M2 microscope (Axio Imager.M2, AxioCam MRm3; Carl Zeiss Microscopy LLC, New York). Reactive astrogliosis was quantified via optical density (OD) analysis and astrocyte cell counting using applications in ZEN software (Carl Zeiss Microscopy LLC). Optical density and cell count analyses were performed on the entirety of the ×40 magnification image (1388 × 1040 pixels: 355.26 μm × 266.19 μm). To obtain OD measurements, we first determined the optical intensity (OI) of each image using the ZEN software. Background OI was measured in an area of the brain (cerebral cortex) where there were no astrocytes. Optical density was then calculated using the equation: log_10_(OI_background_/OI_total_). Astrocyte counting was performed manually on the same images and OD/cell was determined from these values. Altogether, five (n = 5) mice per experimental group were analyzed. For each mouse, the average of three sections per brain with three images per section was calculated. For quantification of the expansion of astrogliosis into the cortex, intensity was measured through a gradient of identical regions of interest (10 072.96 μm^2^) and the length measured (see Figure 5A; n = 5 mice per diet and genotype group, and 3‐7 distinct cortex sections were measured per mouse; gradient measured in duplicate).

### 2.5 Statistical analyses

Group data are presented as mean ± SEM. Group comparisons were performed by *t* test or two‐way analysis of variance (ANOVA) with Tukey's correction for multiple comparisons. ANOVA results are presented to reflect the effect of the diet (*diet*), the genotype (*genotype*), and the presence of an interaction between the diet and genotype variables (*diet* × *genotype*). Statistical significance was set at .05 (GraphPad Prism 6.0).

## 3 RESULTS

### 3.1 Gene expression

Relative mRNA levels of specific genes of interest were examined in *aldh5a1* ^*+/+*^ (wild‐type) and *aldh5a1* ^*−/−*^ (murine SSADHD) mouse brains. Groupings included GABA‐related genes (including *abat*, *aldh5a1*, *gad1*, *glud1*, *gls*, and *glul*) and glutamine transporter genes (including *slc1a5*, *slc7a5*, *slc7a6*, *slc38a1*, *slc38a2*, *slc38a3*, *slc38a5*, and *slc38a7*) (see Figure 1). For the analyses, relative expression (RE; 2^−ΔΔCT^) for each group was compared to the control diet fed wild‐type mouse cohort. For statistical analyses, a two‐way ANOVA was utilized on delta‐cycle threshold (CT) values.

**FIGURE 2:**
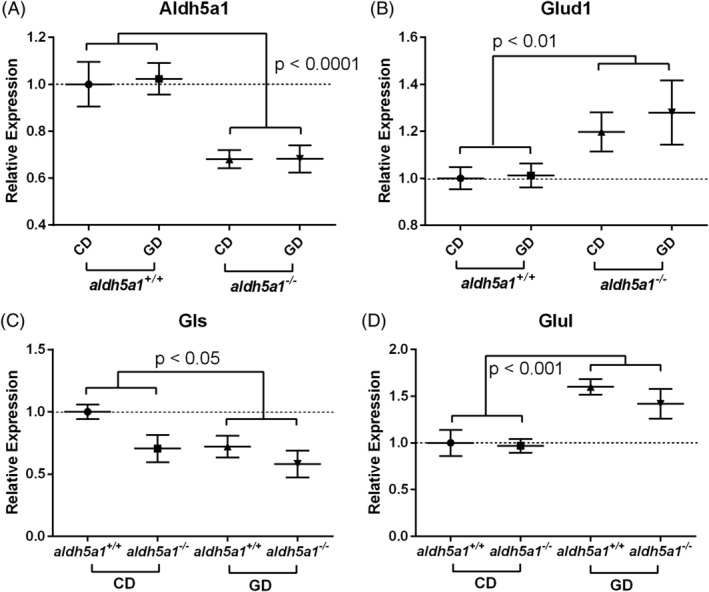
GABA‐ and glutamine‐related gene transcripts. A,B, Genotype differences (*aldh5a1* ^*+/+*^, wild‐type; *aldh5a1* ^*−/−*^, mutants). C,D, Diet differences (CD and GD). *aldh5a1*, aldehyde dehydrogenase family 5, subfamily A1; *glud1*, glutamate dehydrogenase; *gls*, glutaminase; *glul*, glutamate‐ammonia ligase or glutamine synthetase. Statistics analysis of variance (ANOVA) performed as described in Section 2. CD, control diet; GABA, γ‐aminobutyric acid; GD, glutamine diet

The expression of genes involved in GABA and glutamine metabolism is shown in Figure 2. As expected, there was a significant downregulation of *aldh5a1* in *aldh5a1* ^*−/−*^ mouse brains and no diet effect on this gene (RE: *aldh5a1* ^*+/+*^ = 1.0 ± 0.08 and *aldh5a1* ^*−/−*^ = 0.68 ± 0.05; ANOVA: *genotype*: *P* < .0001; *diet*: *P* = .86; *diet* × *genotype*: *P* = .89; Figure 2A). *Glud1* expression was upregulated in *aldh5a1* ^*−/−*^ mice compared to *aldh5a1* ^*+/+*^ mice in both diet groups but there was no difference between diet groups (RE: *aldh5a1* ^*+/+*^ = 1.0 ± 0.05 and *aldh5a1* ^*−/−*^ = 1.2 ± 0.1; ANOVA: *diet*: *P* = .53, *genotype*: *P* < .01, *diet* × *genotype*: *P* = .66; Figure 2B). In contrast, *gls* expression was downregulated in *aldh5a1* ^*−/−*^ mice compared to *aldh5a1* ^*+/+*^ mice and downregulated by glutamine supplementation (RE: *aldh5a1* ^*+/+*^ = 0.86 ± 0.07 and *aldh5a1* ^*−/−*^ = 0.64 ± 0.1; CD = 0.85 ± 0.08 and GD = 0.65 ± 0.09; ANOVA: *diet*: *P* = .04, *genotype*: *P* = .03, *diet* × *genotype*: *P* = .59; Figure 2C). There was a modest but significant change (higher) in the expression of *glul* in glutamine‐fed groups but no genotype difference (RE: CD = 0.98 ± 0.1 and GD = 1.5 ± 0.12; ANOVA: *diet*: *P* < .001, *genotype*: *P* = .45, *diet* × *genotype*: *P* = .66; Figure 2D). For *abat* and *gad1* genes, there were no significant *diet* or *genotype* differences, and no significant *diet* × *genotype* interaction (data not shown).

**FIGURE 3:**
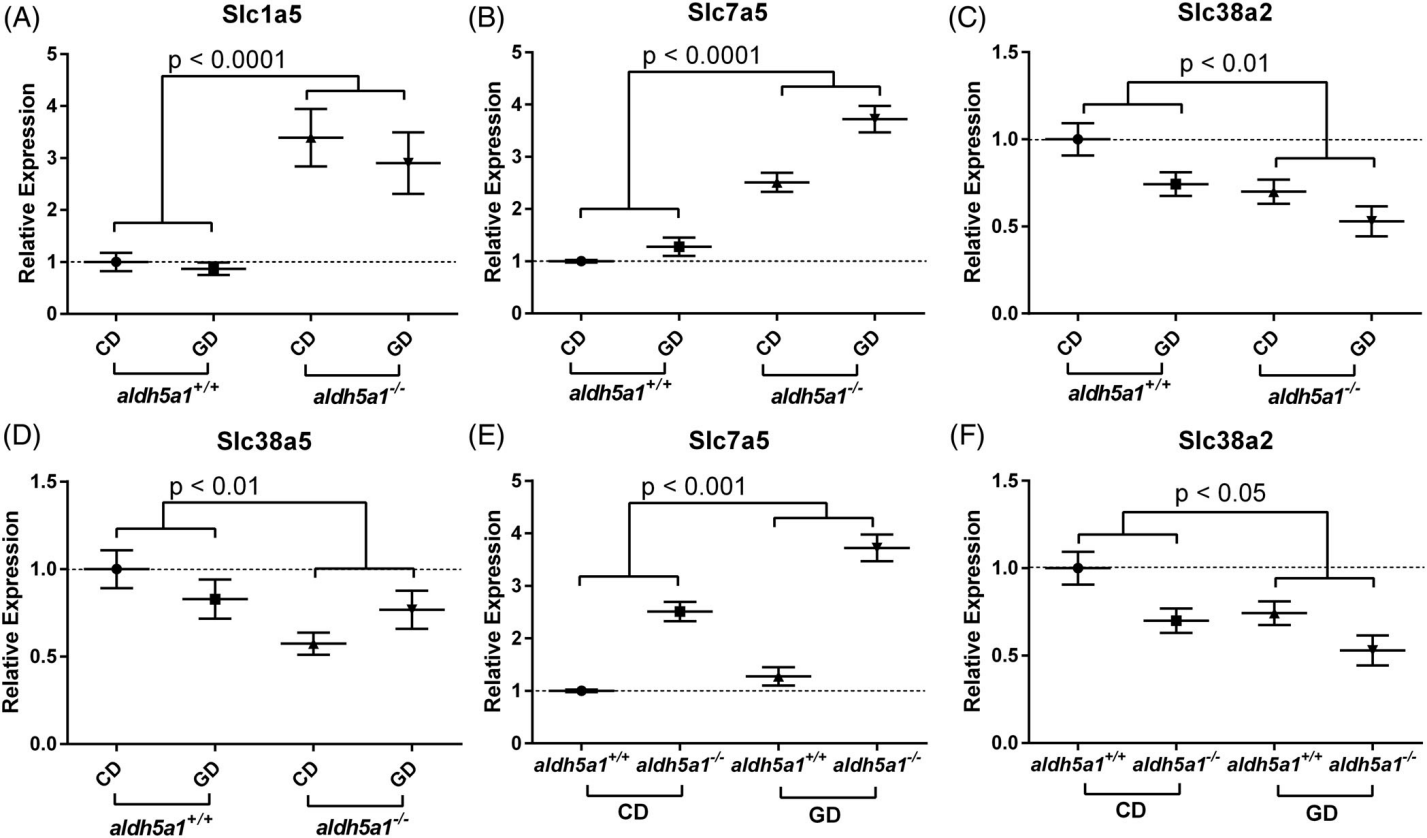
Glutamine transporter gene transcript. A‐D, Genotype differences (*aldh5a1* ^*+/+*^, wild‐type; *aldh5a1* ^*−/−*^, mutants). E,F, Diet differences (CD and GD). *Slc1a5*, solute carrier family 1, member 5; *slc7a5*, solute carrier family 7, member 5; *slc38a2*, solute carrier family 38, member 2. Statistics (ANOVA) performed as described in Section 2. CD, control diet; GD, glutamine diet

The expression of transporter genes is shown in Figure 3. The expression of *slc1a5* was upregulated in *aldh5a1* ^*−/−*^ mice fed either diet (RE: *aldh5a1* ^*+/+*^ = 0.93 ± 0.14 and *aldh5a1* ^*−/−*^ = 3.1 ± 0.57; ANOVA: *genotype*: *P* < .0001; *diet*: *P* = .23; *diet* × *genotype*: *P* = .53; Figure 3A). *Slc7a5* expression was also higher in *aldh5a1* ^*−/−*^ mice compared to wild‐type mice (RE: *aldh5a1* ^+/+^ = 1.1 ± 0.09 and *aldh5a1* ^*−/−*^ = 3.1 ± 0.2; ANOVA *genotype*: *P* < .0001; Figure 3B). However, in contrast to *slc1a5*, it was responsive (higher) in glutamine supplemented groups (RE: CD = 1.8 ± 0.1 and GD = 2.5 ± 0.2; ANOVA *diet*: *P* < .001; *diet* × *genotype*: *P* = .35; Figure 3E). *Slc38a2* gene expression was significantly lower in *aldh5a1* ^*−/−*^ than in wild‐type mice and lower in glutamine‐supplemented mice than in those fed the control diet (RE: *aldh5a1* ^*+/+*^ = 0.87 ± 0.08 and *aldh5a1* ^*−/−*^ = 0.61 ± 0.08; CD = 0.85 ± 0.08 and GD = 0.64 ± 0.08; ANOVA: *diet*: *P* = .02, *genotype*: *P* = .006, *diet* × *genotype*: *P* = .94; Figure 3C,F). *Slc38a5* expression was lower overall in *aldh5a1* ^*−/−*^ mice, although there was a significant *diet* × *genotype* interaction (RE: *aldh5a1* ^*+/+*^ = 0.91 ± 0.11 and *aldh5a1* ^*−/−*^ = 0.67 ± 0.09; ANOVA: *genotype*: *P* = .01, *diet* × *genotype*: *P* = .04; Figure 3D). For the remaining transporters analyzed (*slc7a6*, *slc38a1*, *slc38a3*, and *slc38a7*), there were no significant *diet* or *genotype* differences, and no significant *diet* × *genotype* interaction (data not shown).

### 3.2 Astrogliosis

**FIGURE 4:**
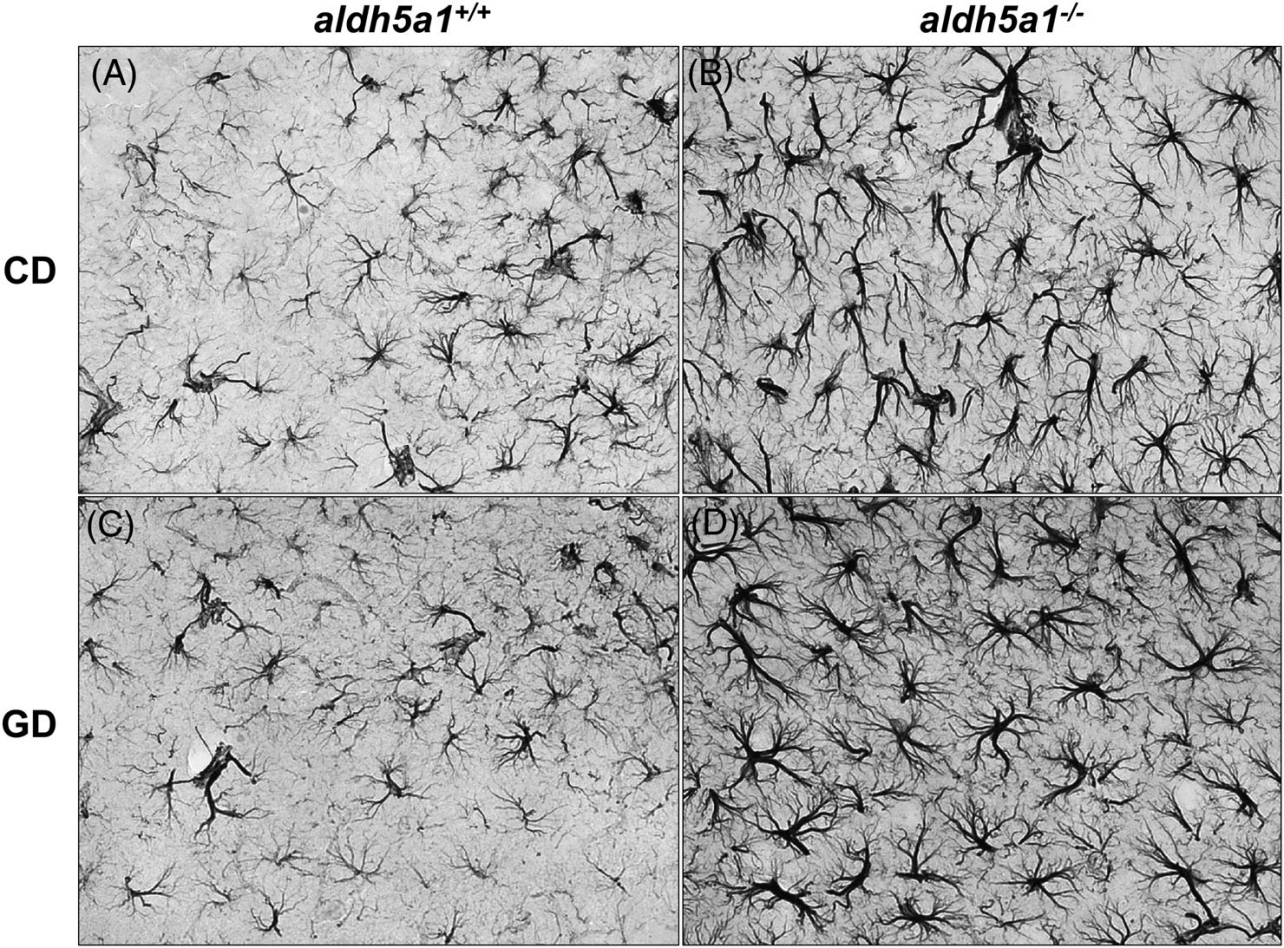
Morphology and density of hippocampal astrocytes in experimental groups. Representative GFAP‐stained sections illustrating astrogliosis in *aldh5a1* ^*−/−*^ mice compared to *aldh5a1* ^*+/+*^ (CD = control diet, panels A and B; GD = glutamine diet; panels C and D). GFAP, glial fibrillary acidic protein

Astrogliosis analyses were performed on GFAP‐stained hippocampal sections. Results are reported in Table 1. There was a significant *genotype* effect on OD, but not on cell count (*aldh5a1* ^*+/+*^ = 0.06 ± 0.005 and *aldh5a1* ^*−/−*^ = 0.11 ± 0.004; ANOVA: *genotype*: *P* < .0001; *diet*: *P* = .73; *diet* × *genotype*: *P* = .48). The *genotype* effect remained statistically significant when calculating the OD per cell ratio, with significantly higher ratios in *aldh5a1* ^*−/−*^ mice than wild‐type mice (*aldh5a1* ^*+/+*^ = 0.99 ± 0.06 and *aldh5a1* ^*−/−*^ = 1.8 ± 0.08; ANOVA: *genotype*: *P* < .0001; *diet*: *P* = .99; *diet* × *genotype*: *P* = .64). Overall, there was no significant *diet* effect and no *diet* × *genotype* interactions. Differences in astrocyte morphology and staining intensity between wild‐type and *aldh5a1* ^*−/−*^ mice are illustrated in Figure 4. After GFAP staining, astrocytes within *aldh5a1* ^*−/−*^ brain sections appeared to have longer and thicker projections, consistent with reactive astrocyte morphology.^11^, ^12^

**TABLE 1:** Astrogliosis quantification in GFAP‐stained brain hippocampal regions

Diet	Genotype	OD		Cell count		OD/cell^a^	
		Mean	SEM	Mean	SEM	Mean	SEM
CD	*+/+*	0.063	0.005	61.7	1.3	1.02	0.08
	*−/−*	0.110	0.005	63.0	1.5	1.76	0.09
GD	*+/+*	0.060	0.003	61.1	1.5	0.96	0.04
	*−/−*	0.120	0.006	64.6	2.0	1.82	0.07

*Note:* Note the higher GFAP‐labeling (OD or OD/cell) in mutants than in wild‐type mice. There was no significant *diet* effect (see Section 3.2 for statistical analysis).

Abbreviations: +/+, *aldh5a1* ^*+/+*^ or wild‐type; −/−, *aldh5a1* ^*−/−*^, mutants; CD, control diet; GD, glutamine diet; GFAP, glial fibrillary acidic protein; OD, optical density.

^a^ Values × 1000.

**FIGURE 5:**
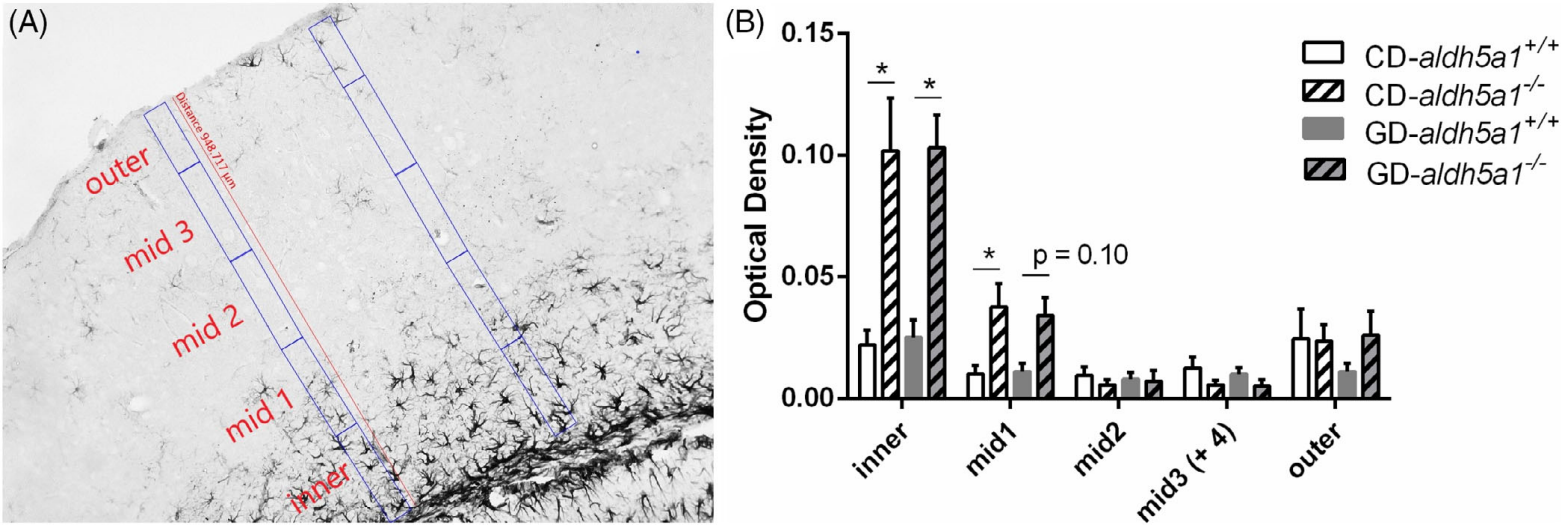
Brain cortical astrogliosis. A, Cortical GFAP labeling measurement zones: inner (closest to corpus callosum), mid 1, mid 2, mid 3, and outer zones (representative cortex section). B, Cortical GFAP labeling (optical density; mean + 1 SE) by zone, diet (CD and GD) and genotype (*aldh5a1* ^*+/+*^, *aldh5a1* ^*−/−*^). Note the significant astrocytic presence in the two zones closest to corpus callosum (inner and mid 1 zones) in *aldh5a1* ^*−/−*^ mice. Statistics were performed as described in Section 2; *P < .05. CD, control diet; GD, glutamine diet; GFAP, glial fibrillary acidic protein

Careful examination of the stained brain sections suggested a greater number of astrocytes in the brain cortex of the *aldh5a1* ^*−/−*^ mice. In order to quantify cortical astroglial expansion, we measured ODs (GFAP staining) across the entire width of the cortex. Five specific zones were selected from the most inner point (corpus callosum) across the entire width of the cortex (zones labeled inner, mid 1, mid 2, mid 3, and outer; see Figure 5A), this to better illustrate astrocytic staining expansion originating from the corpus callosum. Optical density averages for each of the five zones are shown in Figure 5B.

Last, cortex widths and OD/width ratios were calculated to account for a potential increase in OD and astrocyte presence secondary to cortical atrophy. Cortex widths for animals on the control diet were as follows: *aldh5a1* ^*+/+*^ = 1021.9 ± 13.3 μm, *aldh5a1* ^*−/−*^ = 923.3 ± 15.6 μm. Cortex widths for the glutamine‐supplemented mice were: *aldh5a1* ^*+/+*^ = 976.2 ± 20.7 μm and *aldh5a1* ^*−/−*^ = 948.6 ± 17.9 μm. Overall, the data showed a significant difference in cortex width between wild‐type and *aldh5a1* ^*−/−*^ mice, but no difference between CD and GD groups, and no significant (*diet* × *genotype*) interaction (ANOVA: *diet*: *P* = .89, *genotype*: *P* = .013, *diet* × *genotype*: *P* = .14). The calculated OD/width ratios for mice on the control diet were as follows: *aldh5a1* ^*+/+*^ = 0.08 ± 0.01 and *aldh5a1* ^*−/−*^ = 0.19 ± 0.02. Ratios for glutamine‐supplemented mice were *aldh5a1* ^*+/+*^ = 0.07 ± 0.01 and *aldh5a1* ^*−/−*^ = 0.19 ± 0.01. Again, the resulting ANOVA showed a significant genotype effect, but no diet effect and no significant (*diet* × *genotype*) interactions (*diet*: *P* = .70, *genotype*: *P* < .0001, *diet* × *genotype*: *P* = .97).

### 3.3 Glutamine synthetase immunohistochemical analysis

Glutamine synthetase (GLUL) stained cells were manually counted in the cerebral cortex of mice for each group. The results were: CD: *aldh5a1* ^*+/+*^: 58.7 ± 3.8 and *aldh5a1* ^*−/−*^: 43.9 ± 2.7; GD: *aldh5a1* ^*+/+*^: 74.5 ± 5.7 and *aldh5a1* ^*−/−*^: 61.9 ± 4.5 (n = 5 each grouping). Overall, there was a significantly higher number of GLUL‐stained cells in the GD groups compared to CD mice and a trend for a lower number of GLUL‐labeled cells in the cortex of *aldh5a1* ^*−/−*^ mice compared to wild‐type mice and (ANOVA: *diet*: *P* = .037, *genotype*: *P* = .084, *diet* × *genotype*: *P* = .89).

### 3.4 Genetic markers of astrogliosis

**FIGURE 6:**
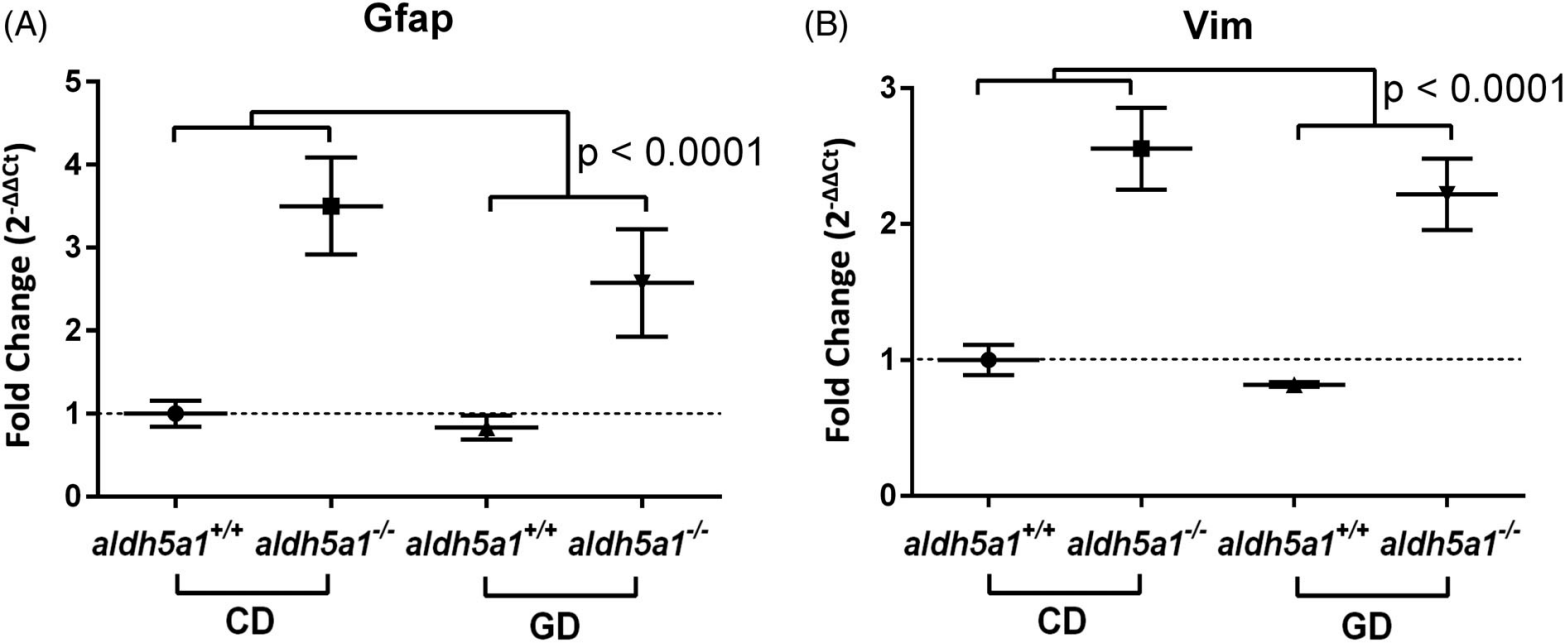
Relative gene expression for markers of astrogliosis. Transcripts significantly altered between genotype (*aldh5a1* ^*+/+*^ = wild‐type; *aldh5a1* ^*−/−*^ = mutants): glial fibrillary acidic protein (*gfap*, A), and vimentin (*vim*, B). Statistics performed as described in Section 2

Astrogliosis was further confirmed by measuring the genetic transcripts of specific markers of astrogliosis, namely *gfap* (glial fibrillary acidic protein), *vim* (vimentin), and *nes* (nestin). There was a significantly greater expression of *gfap* and *vim* in the *aldh5a1* ^*−/−*^ brains compared to the brains of their wild‐type counterparts, but no significant difference between diet groups and no significant (*diet* × *genotype*) interaction (*gfap* RE: *aldh5a1* ^*+/+*^ = 0.91 ± 0.1 and *aldh5a1* ^*−/−*^ = 3.0 ± 0.6; ANOVA: *diet*: *P* = .12, *genotype*: *P* < .0001, *diet* × *genotype*: *P* = .48; *vim* RE: *aldh5a1* ^*+/+*^ = 0.91 ± 0.06 and *aldh5a1* ^*−/−*^ = 2.4 ± 0.3; ANOVA: *diet*: *P* = .11, *genotype*: *P* < .0001, *diet* × *genotype*: *P* = .78) (Figure 6). There were no group differences in *nes* expression.

## 4 DISCUSSION

As we reported previously, maternal glutamine supplementation did not rescue the clinical phenotype of *aldh5a1* ^*−/−*^ mice, a murine model of human SSADHD, and did not improve brain glutamine deficiency, a putative cause for the central nervous system (CNS) manifestations of the disease.^6^ Though supplementation did not improve glutamine deficiency in *aldh5a1* ^*−/−*^ mouse brains, there was evidence of systemic improvement of glutamine metabolism. Specifically, the ratios of measured blood glutamine to either leucine or isoleucine were both raised 2‐fold after glutamine supplementation (Gln/Leu: CD: 1.6 ± 0.2 vs GD: 3.5 ± 0.4; Gln/Ile: CD: 2.2 ± 0.3 vs GD: 4.4 ± 0.5).^6^ The cellular and molecular mechanisms underlying the resistance of the brain to glutamine supplementation had not been investigated but it was possible that such mechanisms could have been triggered to maintain low levels of glutamine in the brain, thus preventing the accumulation of dietary glutamine‐derived neurotransmitters (GABA and glutamate) in a brain already exposed to high levels of neurotransmitters because of ALDH5A1 deficiency. To explore this possibility, we used brain specimens collected as part of the study reported in Reference 6 and measured the expression of several genes involved in GABA metabolism, glutamine synthesis, and glutamine transport. Additionally, because of the prominent role of astrocytes in regulating glutamine metabolism in the brain, we investigated the potential for glutamine supplementation to have impacted astrocyte morphology and their content in glutamine synthetase. Overall, the results of these investigations do not support our hypothesis that the resistance of the aldh5a1‐deficient brain to dietary glutamine supplementation is secondary to cellular and molecular adaptative mechanisms.

We first sought to evaluate the effect of glutamine supplementation on genes implicated in regulating GABA and glutamine homeostasis in the brain. These studies, the first to investigate the molecular impact of dietary glutamine supplementation in SSADHD, showed that supplementation significantly impacted the expression of several genes: *slc7a5* (up), *slc38a2* (down), *glul* (up), and *gls* (down). SLC38A2 transporters are expressed in neurons and play a role in neurotransmitter shuttling in the GABA‐glutamate‐glutamine cycle.^13^ Lower levels of these transcripts with glutamine supplementation may thus represent an adaptative mechanism to dampen the activity GABA‐glutamate‐glutamine cycle in response to greater systemic availability of glutamine because SLC38A5 or system N2 (SN2) transporters are expressed on astrocyte membranes and contribute to glutamine efflux from the astrocyte.^14^, ^15^ The response of *gls* and *glul* to glutamine supplementation, however, is puzzling. We anticipated an increase in *gls* because the gene product, glutaminase, is a glutamine catabolic enzyme, and a decrease in *glul*, because this gene codes for glutamine synthetase. However, our data show the opposite (lower *gls* transcripts, higher *glul* transcripts). One could speculate that the changes in transcript levels compensate for opposite changes in enzyme activity caused by abundance of glutamine. In this hypothetical scenario, for example, glutamine supplementation would downregulate the activity of glutamine synthetase, and this would in turn trigger an increase in *glul* expression. However, if the role of the GABA‐glutamate‐glutamine cycle is to provide excitatory and inhibitory neurotransmitter homeostasis, it is also to protect the CNS against neurotoxic excess of glutamate and ammonium, the natural products of the reaction catalyzed by glutaminase. With this in perspective, one could predict that the excess of glutamine provided by glutamine supplementation would (a) downregulate *gls* expression to maintain glutamate levels within physiological levels and limit the production of ammonium ions, and (b) upregulate *glul* expression to buffer any excess of glutamate. Why then does the combination of glutamine supplementation, decreased *gls* and increased *glul*, not lead to an increase of glutamine concentrations in the CNS of aldh5a1‐deficient mice? One can only speculate that the glutamine‐glutamate balance in the CNS is primarily determined by aldh5a1‐deficiency and ensuing elevated GABA, not by glutamine availability. In mutant mice fed the control diet, CNS GABA levels are significantly higher, and glutamate and glutamate precursor (glutamine) levels are significantly lower than in their wild‐type counterparts (figure 2 in Reference 6). These levels, although abnormal, reflect the “normal” excitatory‐to‐inhibitory neurotransmitter ratio in aldh5a1‐deficiency. Any alteration of this ratio (such as with glutamine supplementation) would trigger compensatory mechanisms to limit glutamine‐derived glutamate and ammonium accumulation and protect the CNS against the toxicity of these metabolites. In this scenario and to speculate further, low CNS glutamine may actually provide protection against excess glutamate in the brain of mutant mice, and glutamine *restriction* rather than *supplementation* may help rescue disease phenotype. In summary, oral glutamine does impact GABA and glutamine brain homeostatic genes. These changes do not provide a coherent picture that could explain the lack of effect on glutamine levels in the *aldh5a1* ^*−/−*^ brain but offer speculative evidence that homeostatic molecular mechanisms protecting the brain from neurotoxic metabolites may be triggered by the dietary intervention.

Our data may not shed conclusive molecular explanations to the resistance of the brain to glutamine supplementation but provide novel molecular insights into the *aldh5a1*‐deficient brain. For instance, we observed a higher expression of *slc7a5* in the mutant brain. *Slc7a5* encodes for LAT1, one of the primary transporters of nearly all neutral essential amino acids to cross the blood‐brain barrier into the brain.^16^, ^17^, ^18^ It is thus possible this transcript is higher in the *aldh5a1* ^*−/−*^ mouse brain because of low brain glutamine levels. Interestingly, the expression of this gene is increased by glutamine supplementation. This suggests that the transport of glutamine by *slc7a5* is not defective in *aldh5a1* ^*−/−*^ mice and its impairment cannot account for central glutamine deficiency in this model.

We also found that *slc1a5* expression is elevated in the *aldh5a1* ^*−/−*^ brain. This observation may be of pathogenic significance. *Slc1a5* encodes for ASCT2 which heterodimerizes with LAT1 to form an obligatory amino acid exchanger.^19^, ^20^, ^21^, ^22^ Together, LAT1 and ASCT2 have been linked to the activation of mammalian target‐of‐rapamycin complex 1 (mTORC1), cell proliferation, and epilepsies.^19^, ^23^, ^24^, ^25^, ^26^ Activation of the mTOR pathway has also been associated with the neuropathological phenotype of tuberous sclerosis complex (TSC), a rare autosomal dominant genetic disease that features astrogliosis and seizures.^27^, ^28^, ^29^ Finally, we reported that mTOR inhibitors such as Torin 1 and Torin 2 increase survival in the *aldh5a1* ^*−/−*^ mouse model.^30^ Taken together, our data and previous reports raise the possibility that central glutamine deficiency may upregulate *slc1a5* and *Slc7a5* gene expression, activate the mTOR pathway, and contribute to astrogliosis and epilepsy, some of the key features of experimental SSADHD.

Astrogliosis has previously been reported in SSADHD mouse brain,^31^ but to our knowledge, the potential impact of glutamine supplementation on astrogliosis has not been studied in this condition. Astrogliosis is a common feature of several brain disorders including Alzheimer's disease, Parkinson's disease, ataxia telangiectasia (A‐T), TSC, glutamine synthetase deficiency, epilepsy, and stroke,^32^ reflecting a cellular reaction (ie, reactive astrocytosis) to a primary insult in the CNS. Our interest in studying the effect of glutamine supplementation on astrogliosis in our murine model of SSADHD stems from evidence that astrogliosis is associated with downregulation of glutamine synthetase and increased neuronal excitability, a potential epileptogenic factor.^33^, ^34^ Also, studies have shown glutamine supplementation is neuroprotective and improves brain tissue inflammation, a trigger for reactive astrogliosis.^35^, ^36^ Our data confirm our earlier reports of significant astrogliosis and glutamine deficiency in aldh5a1‐deficient mice, and indicate a definite trend (*P* = .08) toward *glul* deficiency suggesting that, in this mouse model of SSADHD astrogliosis may be responsible for central glutamine deficiency. Our data further show a significant and positive response of *glul* expression to glutamine supplementation. However, glutamine supplementation did not improve the histological (astrogliosis) phenotype of the mutants and did not improve the mutants' clinical phenotype as observed in the A‐T mouse model.^36^ These contrasting outcomes are difficult to explain. One could speculate that glutamine supplementation provides functional rescue of astrocytic function (as attested by *glul* increase) without decreasing the astrocyte expansion throughout the brain. Alternatively, it might take a longer exposure to glutamine to see improvement of the cellular phenotype (astrocyte expansion) than to see a functional (glul increase) improvement. Such hypotheses unfortunately cannot be tested because of the short lifespan of the mutants. Last, it could be that the underlying metabolic defect (aldh5a1 deficiency with SSA, GABA, and GHB accumulation) is an overriding trigger of reactive astrogliosis. This scenario will need to be validated in future studies where *aldh5a1* deficiency is corrected using either gene editing strategies or enzyme replacement therapy.

Our histological and molecular data may not provide a conclusive explanation to why glutamine supplementation did not rescue the *aldh5a1*‐deficient brain phenotype, but they significantly expand our characterization of astrogliosis in the SSADHD mouse. We observed that *aldh5a1* ^−/−^ astrocytes had significantly stronger GFAP staining (see OD per cell data) than astrocytes in wild‐type mice. We also found that the expression of the gene coding for vimentin (*vim*), a pro‐inflammatory signaling cascade activator^37^, ^38^ was increased in the mutant brain. Taken together, these findings suggest the presence of a strong astrocyte‐mediated inflammatory response in the SSADHD brain and raise the possibility for inflammation to be a significant contributor to SSADHD pathogenesis. Interestingly, there was a trend for a lower expression in these markers of astrogliosis in glutamine‐fed mice. As suggested above, these trends raise the possibility that phenotypic rescue might be possible to demonstrate with a longer exposure to glutamine supplementation, the use of a route of administration of glutamine with greater bioavailability, or simply by increasing the power of our studies with a larger number of animal subjects.

Last, it is worth noting the significant increase in astroglial integration into the sixth layer of the primary somatosensory area of the cerebral cortex of the *aldh5a1* ^*−/−*^ mice compared to their wild‐type littermates. Since astrocyte number interferes with synaptic pruning,^39^ cortical astrogliosis may substantively contribute to the epileptic phenotype of SSADHD as reported for other conditions.^40^, ^41^ Besides this novel observation, many other differences were noted between the wild‐type and *aldh5a1* ^*−/−*^ mouse brain morphologically. We observed differences in the thickness of the cerebral cortex between the two genotypes, with mutants having significantly thinner cortex sectional areas. The smaller cortical thickness of the mutant brain may simply reflect overall stunted growth and general reduction in organ size, or it could be a manifestation of brain atrophy, as reported in SSADHD patients^42^, ^43^, ^44^ and other neurological disorders.^45^

## 5 CONCLUSION

Glutamine‐deficient SSADHD mice were treated with dietary glutamine to rescue central glutamine deficiency, a possible factor in disease pathogenesis. The feeding intervention was not successful despite its significant impact on several genes related to GABA metabolism and glutamine transport in the brain. The study does not provide a conclusive molecular explanation to brain glutamine deficiency and its resistance to treatment. However, it suggests a possible etiological role of reactive astrogliosis and overall brain inflammation.

## CONFLICT OF INTEREST

The authors declare no potential conflict of interest.

## ANIMAL RIGHTS

All institutional and national guidelines for the care and use of laboratory animals were followed.
